# Ultra-Robust Thermoconductive Films Made from Aramid Nanofiber and Boron Nitride Nanosheet for Thermal Management Application

**DOI:** 10.3390/polym13132028

**Published:** 2021-06-22

**Authors:** Li-Hua Zhao, Yun Liao, Li-Chuan Jia, Zhong Wang, Xiao-Long Huang, Wen-Jun Ning, Zong-Xi Zhang, Jun-Wen Ren

**Affiliations:** 1 College of Electrical Engineering, Sichuan University, Chengdu 610065, China; lihuazhao@scu.edu.cn (L.-H.Z.); 2019223035137@stu.scu.edu.cn (Y.L.); lcjia@scu.edu.cn (L.-C.J.); zhongwang@scu.edu.cn (Z.W.); xlhuang@scu.edu.cn (X.-L.H.); ningwj@scu.edu.cn (W.-J.N.); 2 State Grid Sichuan Electric Power Research Institute, State Grid of China, Chengdu 610041, China; 2019223035104@stu.scu.edu.cn

**Keywords:** high thermal conductivity, boron nitride nanosheets, in situ reduction, composites, electrical insulation

## Abstract

The development of highly thermally conductive composites with excellent electrical insulation has attracted extensive attention, which is of great significance to solve the increasingly severe heat concentration issue of electronic equipment. Herein, we report a new strategy to prepare boron nitride nanosheets (BNNSs) via an ion-assisted liquid-phase exfoliation method. Then, silver nanoparticle (AgNP) modified BNNS (BNNS@Ag) was obtained by in situ reduction properties. The exfoliation yield of BNNS was approximately 50% via the ion-assisted liquid-phase exfoliation method. Subsequently, aramid nanofiber (ANF)/BNNS@Ag composites were prepared by vacuum filtration. Owing to the “brick-and-mortar” structure formed inside the composite and the adhesion of AgNP, the interfacial thermal resistance was effectively reduced. Therefore, the in-plane thermal conductivity of ANF/BNNS@Ag composites was as high as 11.51 W m^−1^ K^−1^, which was 233.27% higher than that of pure ANF (3.45 W m^−1^ K^−1^). The addition of BNNS@Ag maintained tensile properties (tensile strength of 129.14 MPa). Moreover, the ANF/BNNS@Ag films also had good dielectric properties and the dielectric constant was below 2.5 (10^3^ Hz). Hence, the ANF/BNNS@Ag composite shows excellent thermal management performance, and the electrical insulation and mechanical properties of the matrix are retained, indicating its potential application prospects in high pressure and high temperature application environments.

## 1. Introduction

With the development of electronic equipment toward miniaturization, high power, and function centralization, new requirements are being put forward for high-performance thermal management materials (TMMs) [1,2]. Thermally conductive composites used as TMMs require good thermal conductivity but also have the advantages of light weight, low cost, and good processing performance in most applications. Polymers are ideal candidates. However, the primary reason for restricting its use in the field of heat dissipation is that polymers generally have a low thermal conductivity (0.2–0.5 W m^−1^ K^−1^) [3,4]. Therefore, a variety of thermally conductive fillers, such as graphene, hexagonal boron nitride (h-BN), aluminum nitride, aluminum oxide, and silicon carbide are usually used to improve the thermal conductivity of polymer composites and solve the problem of heat dissipation [5,6,7,8,9,10].

In certain applications, TMMs are also required to have excellent electrical insulation [11]. Among all of fillers, boron nitride nanosheet (BNNS), also known as “white graphene”, has attracted extensive attention due to its ultrahigh thermal conductivity (200–2000 W m^−1^ K^−1^) and the excellent electrical insulation (5–6 eV band gap) [12,13]. Interestingly, it also has the excellent electrical insulation [14], high chemical stability, and high thermal stability [15,16]. These properties make BNNS a promising filler for TMMs. However, the preparation of BNNSs is more complex than graphene, due to the lip-lip interaction between layers of h-BN, resulting in stronger interlayer forces and shorter interlayer distances [17,18,19]. The exfoliation difficulty of BNNS limits its application to a certain extent. To date, several studies on exfoliation of boron nitride have been conducted. The preparation of BNNS can be divided into a “top-down” exfoliation method and a “bottom-up” synthesis method [20]. Chemical vapor deposition is a typical synthetic method. Li et al. formed BNNS on the solid surface in a nitrogen environment by using solid borates as precursors [18]. The advantage of this technology is that there are many kinds of borates as precursors, so the raw materials are easy to obtain. However, the common problem of chemical vapor deposition is that the precisely localized injection of gas precursors into the designated places of the growth substrate is still uncontrollable. Physical vapor deposition and liquid phase exfoliation are common in the exfoliation method. Muhammad et al. deposited polycrystalline BNNS at 350 °C at 0.2 Pa, followed by single crystal BNNS at 26 Pa in a hydrogen environment [21]. Unfortunately, the harsh experimental conditions, especially the dangers of hydrogen, have limited the use of this technology. By adding h-BN powder into the mixed solvent of ammonia and isopropyl alcohol, Wang et al. obtained BNNS with a complete crystal structure after water bath ultrasound [22]. Although they relied on the theory that the binding of amino molecules to boron electrons would improve the exfoliation effect, the increase in yield did not seem to be mentioned. From previous reports, the solvent used in the liquid phase exfoliation method is particularly critical [22,23]. In addition, according to report of Wang et al., Na^+^ and K^+^ produced by NaOH and KOH in the molten state also have a positive effect on exfoliation, although it may be dangerous [17]. At present, how to efficiently prepare BNNS is still a challenging issue.

In addition to the issue of preparation of BNNS, the inevitable interfacial thermal resistance between BNNS and the matrix also significantly hinders the improvement of thermal conductivity of the composites [24,25,26,27]. The interfacial thermal resistance is mainly caused by the poor contact between the fillers and the matrix [28,29], and the synergistic effect of modified fillers or hybrid fillers can effectively reduce the interfacial thermal resistance [29,30,31,32]. According to the research of Wang et al., an effective heat transfer bridge was formed between silver nanoparticles (AgNP) and BNNS, and the interfacial thermal resistance was partially replaced by the lower contact thermal resistance, thus further improving the thermal conductivity [33,34]. Therefore, AgNP has a good prospect in enhancing the interaction between fillers [35].

Based on the aforementioned viewpoints, in this study, we simplified the process, and proposed a new method that can generate AgNP modified BNNS (BNNS@Ag) while efficiently exfoliating boron nitride. This article examines the way in which the influence of solvent ratio and ion type and concentration on the liquid-phase exfoliation, further in situ reduction to generate BNNS@Ag, and verify that the generated BNNS@Ag can reduce the interfacial thermal resistance, and improve the thermal conductivity of the polymer. The first step in this work was to explore the effects of different ratios of water and isopropanol, and different citrate types and concentrations on the exfoliation yield. Then BNNS@Ag was synthesized by the in situ reduction of citrate, which was added into aramid nanofiber (ANF) to prepare ANF/BNNS@Ag composites with a “brick and mortar” structure. The result shows that the thermal conductivity of ANF/BNNS@Ag is significantly higher than that of pure ANF material, ANF/h-BN composite, and ANF/BNNS composite. In addition, the mechanical and electrical properties of the composites were studied, and it was found that the tensile strength and electrical insulation of the composites were not significantly damaged.

## 2. Materials and Methods

### 2.1. Materials

Raw h-BN flakes (average size of 10 μm, 99.5% purity) were purchased from Qinhuangdao Eno High Tech Material Development, Qinhuangdao, China. Trisodium citrate, lithium citrate, and potassium citrate were purchased from Shanghai Aladdin Biochemical, China. The Kevlar^®^ 29 fibers were purchased from DuPont. Silver nitrate (AgNO_3_), trisodium hydroxide (NaOH), isopropanol (IPA), potassium hydroxide (KOH), dimethyl sulfoxide (DMSO), ethanol alcohol, and deionized water (DI H_2_O) were obtained from Chengdu Kelong Chemical Reagent Co., Ltd., Chengdu, China, and used as received.

### 2.2. Preparation of BNNS, BNNS@Ag, and ANF

BNNS were prepared via the ion-assisted liquid-phase exfoliation method. Firstly, 1 g of h-BN and 1 g of trisodium citrate were added into a 100 mL mixture of H_2_O and IPA (4:1) and then treated adequately with a cutting-edge ultrasound (750W, Ningbo Scientz Biotechnology Co., Ltd., Ningbo, China) for 30 min. Subsequently, the mixture was treated by ultrasonication for 6 h, and a hydrothermal reaction was carried out at 180 °C for 6 h. The solution were removed after cooling in the air. After that, the solution was centrifuged at 1000 rpm for 20 min to remove the exfoliated h-BN, and the supernatant was washed three times by vacuum filter to remove trisodium citrate. The BNNS solution was obtained after the remaining filter cake was dissolved in 220 mL DI H_2_O. The above steps were repeated with different ratios of H_2_O and IPA (5:0, 3:2, 2:3, 1:4, and 0:5), and different citrate types (lithium citrate, potassium citrate, and no citrate).

In addition to exfoliation of h-BN assisted by citrate as described above, the in situ reduction property of citrate was used to deposit AgNP on the BNNS surface. The silver nitrate aqueous solution (50 mmol/L, 11.765 mL) was dropped into the BNNS solution containing trisodium citrate. The mixture was stirred at 60 °C at 1000 rpm for 24 h. Then it was washed three times by vacuum filtration to remove the trisodium citrate. At last, the BNNS with AgNP adsorption on the surface were obtained after the remaining filter cake was dissolved in 220 mL DI H_2_O, namely BNNS@Ag.

The ANF was obtained by the method of deprotonation. First 1.6 g of Kevlar^®^ 29 fibers were cut into short strands less than 1 cm in length. Subsequently Kevlar^®^ 29 fibers were soaked in acetone and ultrasonized for 24 h to remove impurities in an ultrasonic cleaning machine (KH5200DE, Ningbo Scientz Biotechnology Co., Ltd., Ningbo, China). Kevlar^®^ 29 fibers was dried under vacuum at 45 °C for 24 h after the acetone was filtered out. Then 1.6 g of Kevlar^®^ 29 fibers, 2.4 g of KOH, 12.8 mL of H_2_O, and 320 mL of DMSO were added in a round-bottom flask with magnetic stirring at 1000 rpm for 2 weeks, and a fluorescent red ANF/DMSO dispersion was obtained. The dispersion was injected into 400 mL of deionized H_2_O and stirred vigorously, and then a yellowish colloid formed. The colloid was collected through filtration, and then ethyl alcohol was added and washed more than 6 times to remove alkali entirely. At last, deionized water was added to make an ANF/H_2_O dispersion with an ANF content of about 2 mg/mL, and the ANF solution was obtained.

### 2.3. Preparation of Thermal Conductive Composite Films

The ANF/BNNS@Ag composite films were prepared using the vacuum-assisted filtration method. First, the fillers were added into the ANF solution. The mixture was sheared strongly with a high-speed shear emulsifier (13,000 rpm, IKA, Staufen, Germany) for 6 h, and then the rudiment of the composite films was obtained by vacuum filtration. Finally, the ANF/BNNS@Ag composite films were prepared by hot pressing at 100 °C for 10 min and dried under vacuum at 45 °C for 12 h (DZF-6090, Bluepard, Shanghai, China). By setting the mass ratio of BNNS@Ag to the mixture to 10%, 20%, 30%, and 40%, a series of composite films with different concentrations were obtained. The pure ANF, ANF/h-BN, and ANF/BNNS composite films were prepared by the same procedure of ANF/BNNS@Ag composite films.

### 2.4. Characterization

The microstructures and morphologies of the h-BN, BNNS, BNNS@Ag, and fractured surfaces of films were investigated via scanning electron microscopy (SEM, Nova NanoSEM 450, 5 kV, FEI, Hillsboro, OR, USA), and the Kevlar^®^ 29 fibers were investigated via SEM (SU8010, 1 kV, HITACHI, Tokyo, Japan). Transmission electron microscopy (TEM) images of BNNS, BNNS@Ag, and ANF were taken using a JEOL2100F transmission electron microscope at 200 kV (Tokyo, Japan), and the atomic force microscopy (AFM) measurement of BNNS was carried out using a Bruker Dimension Icon instrument (Billerica, MA, USA). Raman spectra of the h-BN and the BNNS were measured from 1000 to 2000 cm^−1^ using a Horiba LabRAM HR micro-Raman system (Horiba, Kyoto, Japan) equipped with a 633 nm laser excitation. X-ray diffraction (XRD) patterns of h-BN, BNNS, and BNNS@Ag were performed on a PANalytical X’Pert Pro diffractometer (Almelo, The Netherland) with Cu Kα radiation (λ = 0.154 nm) at 25 °C and scanning from 5° to 90° at a speed of 10°/min. The yield (Y) of BNNS was calculated according to Equation (1):

(1) $$Y=\frac{W_{\mathrm{BNNS}}}{W_{h-\mathrm{BN}}}\times 100\%\;$$

where W_BNNS_ is the weight of the obtained BNNS after drying into powder, and W_h-BN_ is the weight of the initial h-BN. The elemental composition of each sample was detected via X-ray photoelectron spectroscopy (XPS) on a Thermo Scientific K-Alpha (Thermo Fisher, Waltham, MA, USA) with Al Ka (mono, 1486.6 eV, voltage of 15 kV). Fourier transform infrared (FT–IR) spectra were collected using a Nicolet-5700 Fourier transform infrared spectrometer (Thermo Nicolet Corporation, Madison, SD, USA). Thermogravimetric analysis (TGA) of the BNNS, the BNNS@Ag, and composite films was conducted using a Mettler Toledo thermogravimetric analyzer (Barcelona, Spain) from 30 °C to 800 °C at a heating rate of 10 °C/min under a N_2_ flow (20 mL/min). The in-plane thermal conductivity (λ) was calculated via Equation (2) [36,37] as follows:

(2) $$\lambda =\alpha C_{p}\rho$$

where *ρ* is the density of composite film, *Cp* is the specific heat capacity of composite films, and *α* is the thermal diffusivity of the composite films. The *Cp* values of composite films were measured using differential scanning calorimetry (DSC, TA 2100, PerkinElmer, Waltham, MA, USA) with the Sapphire method. The in-plane *α* of the composite films were investigated using a NETZSCH LFA 467 Laser Flash Apparatus (Netzsch, Selb, Germany). The densities (*ρ*) of the composite films were calculated using the equation *ρ* = *W*/*V*, where *W* and *V* are the weight and volume of the composite films, respectively. Differential scanning calorimetry (DSC) of composites was performed on a TA 2100 calorimeter with a heating rate of 5 °C/min under an N_2_ flow (20 mL/min). The dielectric responses of the composites were analyzed using an HP 4194A impedance analyzer (Agilent Technologies, Santa Clara, CA, USA) over the frequency range of 10^0^ to 10^6^ Hz. The electrical volume resistivities of the composite films were tested using 6587 Picoammeter (Tektronix, Beaverton, OR, USA) at room temperature. The variation of surface temperature of the polymer composites under sustained heating was recorded by an infrared thermograph camera (Testo 890, Lenzkirch, Germany). The images of the h-BN, the BNNS, and the BNNS@Ag dispersions and their Tyndall effects were captured by a HUAWEI P40 (Shenzhen, China).

## 3. Results and Discussion

### 3.1. The Efficient Exfoliation of BNNS@Ag

**Figure 1:**
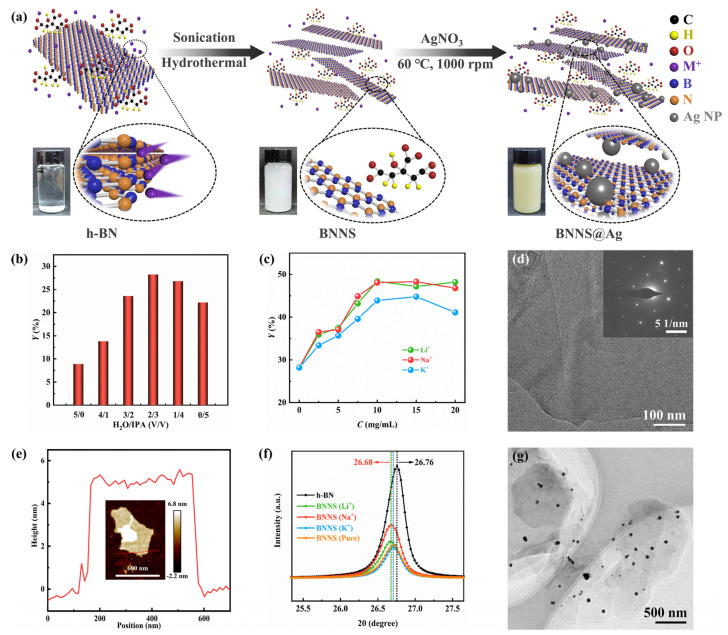
Fabrication of BNNS@Ag. (**a**) Schematic diagram of BNNS fabricated via the liquid-phase exfoliation assisted by alkali metal ions, and in situ synthesis of AgNP on BNNS by citrate. The exfoliated yields of BNNS (**b**) in different ratios of H_2_O and IPA, and (**c**) with different types of alkali metal ions. (**d**) Corresponding electron diffraction pattern of BNNS. (**e**) AFM image of BNNS and corresponding height profiles of BNNS. (**f**) XRD patterns of h-BN, BNNS exfoliated by Li^+^, Na^+^, K^+^, and pure solvent. (**g**) TEM image of BNNS@Ag.

In the top-down approach, the liquid-phase exfoliation method was the most widely used because of its low cost, simple process, and the possibility of further improvement [38,39,40,41]. In Figure 1a, the process of exfoliation of h-BN into BNNS and attachment of AgNP on its surface is shown. First, based on the typical liquid phase exfoliation method using ultrasonic and hydrothermal reaction, the cationic intercalation was used to assist exfoliating BNNS. The h-BN that was used had a surface pretreatment with NaOH, which could weaken the interaction between adjacent h-BN layers [17]. The dispersion of BNNS in IPA was significantly better than that in H_2_O. This was because the surface energy of IPA was matched to the surface tension of h-BN, and the solvent whose polarity was well matched with the surface energy of h-BN could reduce the exfoliation energy [19,42]. Nonetheless, the solubility of alkali metal salts in water was better than that of IPA, so a certain amount of H_2_O was still needed. Therefore, in this work, the exfoliation of BNNS at different ratios of H_2_O and IPA was explored to obtain the optimal exfoliation yield. As shown in Figure 1b, without adding alkali metal salt, we noticed that the yield gradually increased from H_2_O:IPA = 5:0 and reached the maximum value of nearly 30% when the ratio was 2:3, and then the yield showed a slight downward trend. The reason for the increasing trend from 5:0 to 2:3 was that the BNNS dispersed better due to the addition of IPA, and the ultrasonic cavitation effect of IPA was better than that of H_2_O because the surface tension of IPA (21.7 dyn/cm) was less than that of H_2_O (72.7 dyn/cm) [19]. A possible explanation for the downward trend of yield after 2:3 was that when IPA:H_2_O = 2:3, the mixed solvent had the best Hansen solubility parameters and the most suitable solvent viscosity, which was beneficial to the dispersion stability of BNNS [38].

On the other hand, as noted by Wang et al. [40] and Wang et al. [19], different kinds of ions intercalated also had a certain influence on the exfoliation. Therefore, the influence of different ion types and ion concentrations on the exfoliation yield of BNNS was also explored in the work. The promoting effect of ions on exfoliation was reflected in that, when ions were inserted into the interlamellar space of h-BN, ions first adsorbed on the surface of h-BN, and then diffused into the space between the lattices of adjacent h-BN, finally causing the h-BN at the edge and top to curl or wrinkle [17]. When H_2_O:IPA = 2:3, the exfoliation yields of Li^+^ and Na^+^ were significantly better than those of K^+^ (Figure 1c). This could be due to the ionic radii of Li^+^ and Na^+^ being smaller than those of K^+^, and the cations with smaller radii were more likely to insert between layers of h-BN for exfoliation [40]. Moreover, it could be clearly seen that with the increase of ion concentration, the exfoliation yield gradually increased. Then after the concentration reached 10 mg/mL, the yield slightly decreased. Too high a concentration of ions could not further enter between layers. Instead, the concentration of ions was too high around h-BN, which inhibited the exfoliation of h-BN, resulting in a small decrease in the yield. Figure S1b (Supplementary Materials) showed the exfoliated BNNS in mixed solvents with different H_2_O and IPA ratios, and with different ion types. Meanwhile, the h-BN and BNNS prepared by various cations were analyzed by XRD (Supplementary Materials, Figure S3a). The characteristic peaks of the four types of BNNS corresponded with the characteristic peaks of h-BN [43,44], indicating that the crystal structure of BNNS remained intact. It was noted that the peak values of all kinds of BNNS were significantly smaller than the peak values of h-BN, and the positions of the peaks were slightly shifted (Figure 1f), indicating the reduction of interlayer distance and the weakening of interlayer forces, which proved that h-BN had been successfully prepared into BNNS. The peak shift of Li^+^ and Na^+^ was 26.68, which was more than that of K^+^ and pure solvent. Moreover, based on Figure 1c, we believed that the exfoliation efficiency of Li^+^ and Na^+^ was superior to that of K^+^ and pure solvent.

In Supplementary Materials, Figure S2a, it can be seen that h-BN had almost no transmittance and presented a dense massive structure with multiple layers piled, and the width of h-BN on its surface was about 1–2 μm. It could be seen that there were at least 30 layers of h-BN stacked together (Supplementary Materials, Figure S2b). Previous reports had shown that the thickness of single-layer BNNS was about 3.33 Å [19,45], so the thickness of h-BN there was at least 99.9 Å. In contrast, the SEM image of BNNS (Supplementary Materials, Figure S2c) showed that the BNNS were translucent and relatively smooth, presenting an ultra-thin sheet structure with few layers stacked, 0.2–0.8 μm in size, and fewer than 10 layers. Further observation of the TEM image of BNNS showed that the transmittance of BNNS was very high, and some areas were nearly transparent (Supplementary Materials, Figure S2d). It could be seen that the BNNS had less than five layers of accumulation, or even a single layer. The black part of the edge area was the fold caused by the thin BNNS. In addition, as shown in Figure 1d, the electron diffraction pattern of BNNS clearly showed a typical hexagonal symmetry highly crystalline structure pattern of BNNS, which again proved that BNNS retains the integrity of their crystal structure. In Figure 1e, the surface morphology and thickness of BNNS were further shown. The image shows a 15 layer BNNS with a thickness of approximately 5 nm, and a transverse dimension of approximately 500 nm. There were differences with TEM images, which could be attributed to the unevenness of the thickness of the prepared BNNS, and the difference in the test area. Furthermore, from a macro perspective, in the mixed solvent of H_2_O and IPA, the dispersion of BNNS was significantly better than that of h-BN (Supplementary Materials, Figure S1a), which further proved that the exfoliation of BNNS was successful. In addition, the Raman spectra of h-BN and BNNS were shown in Figure S3b (Supplementary Materials). The E2_g_ peak of the BNNS showed a slight blue shift, indicating a decrease in the number of layers, while the Raman peak strength of BNNS was weaker than that of h-BN powders, which was attributed to the weakened interlayer interaction, further confirming that the h-BN powders were exfoliated into fewer layers of BNNS, which was consistent with the results of SEM and TEM. Therefore, it was reasonable to believe that BNNS with fewer layers, smooth surface, good dispersion, and intact structure could be obtained by using H_2_O:IPA = 2:3 mixed solvent, and inserting Li^+^ or Na^+^ with a concentration of 10 mg/mL into the h-BN interlayer assisted exfoliation.

Citrate is a commonly used reductant in metal reduction system [46,47,48]. As shown in the second step of Figure 1a, due to the property of in situ reduction, citrate lost an electron and generated CO_2_ and AgNP [49]. Then BNNS@Ag was obtained via the adhesion of AgNP [47,50]. It could be seen from XPS that Ag 3d had two obvious peaks located at 367.8 eV and 373.6 eV, respectively (Supplementary Materials, Figure S5), which could indicate that Ag had been successfully generated. According to previous reports, the peaks of single AgNP should be 368.5 eV and 374.4 eV, and the different peaks of the two were 0.7 eV and 0.8 eV [33], respectively, which proved from the side that AgNP existed in the form of physical adsorption or electrostatic adsorption. In addition, according to the analysis in Figure 1a, it could be seen that the color of BNNS was milky white, while the color of BNNS@Ag was brownish yellow, which further proved that AgNP had been formed, resulting in the color change of the solution.

As can be seen in Figure 1g, the size of the generated AgNP was between 20 and 60 nm. Note that all the AgNP in Figure 1g were randomly distributed on BNNS, and no single AgNPs appeared in the area without BNNS, which proved that AgNPs had been anchored on the surface of BNNS [51,52]. This was further confirmed by SEM image (Supplementary Materials, Figure S4a). Moreover, as can be seen from Figure S4c,d (Supplementary Materials), the XRD images of BNNS@Ag showed new characteristic peaks at 2θ = 38.11°, 64.38°, and 77.28°, in addition to the characteristic peaks of BNNS in Figure S3a (Supplementary Materials), corresponding to the planes of AgNP (111), (220), and (311). Comparison of the finding with those of other studies confirmed that AgNP had been physically deposited on the BNNS surface by Ag [33,34,53,54], that is, AgNP had been successfully modified on the BNNS surface. In addition, it could be seen from Figure 1g and Figure S4a (Supplementary Materials) that the morphology of BNNS itself was well maintained without the phenomenon of multilayer accumulation, indicating that the adhesion of AgNP did not lead to the re-agglomeration of BNNS. Specifically, it can be seen from Figure S4b (Supplementary Materials) that the edge of BNNS@Ag was still wrinkled, indicating that BNNS@Ag still maintained the characteristics of a few-layer structure. As shown in Figure S6a (Supplementary Materials), the FT-IR spectra of BNNS@Ag were very similar to that of BNNS. The former presented characteristic peaks at 1348 cm^−1^ and 788 cm^−1^, while the latter presented characteristic peaks at 1342 cm^−1^ and 772 cm^−1^, which also accorded with other reports [36,55,56]. The two peaks contained the vibrations of B−N stretching and B−N bending, respectively, while the slight blue shift of BNNS@Ag relative to BNNS could be attributed to the slight change in lattice vibration caused by the successful adsorption of AgNP on the surface of BNNS. Moreover, no other characteristic peaks were observed from the FT-IR spectra, indicating that no other groups were introduced in this experiment. When heated to 800 °C, the mass of BNNS and BNNS@Ag did not change significantly, and the mass losses were 0.11 wt.%, 1.04 wt.%, and 0.70 wt.%, respectively (Supplementary Materials, Figure S6b), indicating that the thermal stability of BNNS@Ag was still good, consistent with that of BNNS. In addition, it can be seen from Figure S7 (Supplementary Materials) that BNNS@Ag and BNNS had the same Tyndall effect, which further confirmed the good dispersion of BNNS@Ag.

Per the discussion mentioned above, control experiments were carried out in this study for different H_2_O and IPA ratio mixed solvents and different kinds of alkali metal ions. The conclusion was that when H_2_O:IPA = 2:3, and the ion type was Li^+^ and Na^+^, the exfoliation efficiency that h-BN turned into BNNS was the highest. The prepared BNNS had the characteristics of ultra-thin, smooth surface, good dispersion, and hard agglomeration. After that, citrate was used to reduce Ag^+^ to Ag in situ, and AgNP was formed by adsorption on the surface of BNNS, thereby obtaining BNNS@Ag. The obtained BNNS@Ag still had the above characteristics of BNNS, and the size of AgNP adsorbed on BNNS was small, and it was evenly distributed on the surface of BNNS.

### 3.2. Deprotonation Process of ANF and Preparation of Composite Films

**Figure 2:**
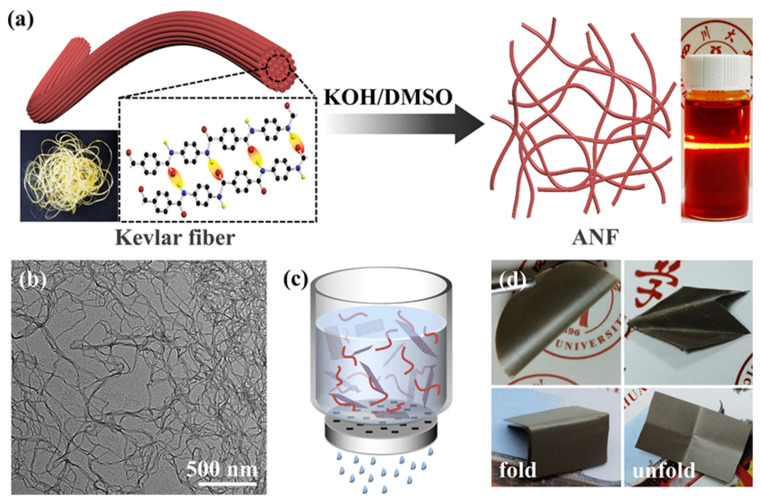
Fabrication of ANF and ANF/BNNS@Ag composite films. (**a**) Schematic process of the preparation of ANF by dissolving Kevlar^®^ 29 fibers in DMSO with the assistance of KOH. (**b**) TEM image of the ANF. (**c**) Schematic diagram of vacuum filter device for preparing thermally conductive composite films. (**d**) The photographs of mechanical flexibility of the ANF/BNNS@Ag thermally conductive composite film.

The preparation process of ANF is shown in Figure 2a. Kevlar yarn was a smooth, yellowish yarn, and each molecular chain of Kevlar yarn was interconnected with other molecular chains by hydrogen bonds [57,58,59]. From the SEM image of Kevlar yarn (Supplementary Materials, Figure S8), it can be seen that ANF had a very high aspect ratio. The original Kevlar yarn was about 10–15 μm in diameter (Supplementary Materials, Figure S9b), consistent with the literature [60,61,62], with a slight granular surface protuberance, which was caused by soaking in acetone and further cleaning by ultrasound before dispersing in DMSO solution. In order to break this connection, the deprotonation of amide groups on ANF was needed [62,63,64]. This experiment adopted the method of chemical separation to prepare ANF in DMSO with the assistance of KOH [57,65,66]. Usually, the ratio of KOH to Kevlar yarn is 2:3 [59]. Moreover, after the addition of Kevlar yarn and KOH, and before the addition of DMSO, an appropriate amount of H_2_O should be added. The reason was that, as reported by Bruch et al. in 1990, the presence of proton donors such as alcohols or water can reduce the viscosity of ANF in DMSO, which means that the proton of ANF is easier to lose, that is, the deprotonation process is accelerated, and it is also conducive to the generation of high concentration ANF/DMSO solution, and then shortens the preparation cycle [67]. Secondly, the solubility of KOH in DMSO is poor, so it is necessary to use a certain amount of H_2_O to dissolve KOH in advance to obtain free OH^−^. Therefore, as reported in the literature [62], the volume ratio of H_2_O and DMSO used in this experiment was 1:25. At this ratio, ANF could be uniformly dissolved in DMSO.

The ANF/DMSO solution was prepared after the hydrogen bonds were broken between the fibers. The solution was a clear dark red solution, and the solution exhibited a typical Tyndall effect, which proved the colloidal characteristic of the dispersion. A TEM image of ANF is shown in Figure 2b, and it was noticed that the ANF were uniform in appearance with specific long and entangled morphologies. Additionally, the TEM image exhibited a clear network-like structure with a single fiber diameter of 30–60 nm, and a length of more than dozens of microns, which was in accordance with the previous reports [68,69]. Since abstraction of hydrogen from amide groups, and the constitutive fibers remained intact, the bulk macroscale fiber structure was destroyed into nanofibers and formed a network distribution. Moreover, the electrostatic repulsion between the negatively charged molecular chains was also in favor of the formation of ANF. At last, ANF stably existed in the DMSO under the balance of electrostatic repulsion, π-π bonds, and van der Waals forces [57]. It is worth noting that ANF/DMSO mixed solution was sensitive to moisture even in the air, so that the thin film was formed on the surface of the solution.

Because of the loss of the hydrogen, the molecular structure of the Kevlar yarn was imperfect, which was not what we wanted. Therefore, water was used as proton donor to restore its molecular structure. After adding ANF/DMSO mixed solution into H_2_O and washing it repeatedly, an insoluble colloid substance was obtained (Supplementary Materials, Figure S9a). The role of repeated washing was to wash away DMSO and K^+^, especially K^+^. If there was too much residual K^+^, the electrical insulation performance of the composite material would be seriously affected. The ANF/DMSO mixture formed a wormlike colloid immediately after being added to H_2_O, which was white and opaque after repeated washing. After adding BNNS@Ag (Supplementary Materials, Figure S9b) and shearing at high speed, an ANF/BNNS@Ag mixed solution was obtained (Figure S9c), and the solution was light gray. ANF and BNNS@Ag were dispersed in water, and independent of each other (Supplementary Materials, Figure S9d). The appearance of pure ANF, ANF/h-BN, ANF/BNNS, and ANF/BNNS@Ag thermally conductive composite films obtained by vacuum filtration (Supplementary Materials, Figure 2c) is as shown in Supplementary Materials, Figure S10. It can be seen that pure ANF film was translucent yellow film, while ANF/h-BN, ANF/BNNS, and ANF/BNNS@Ag films were opaque primrose yellow film, milky white film, and dark gray film, respectively. The surfaces of the four kinds of films were smooth, without cracks. Figure 2d presents flexibility of the ANF/BNNS@Ag thermally conductive composite film. ANF/BNNS@Ag film demonstrated restorability after folding without cracks and could even be folded into a paper airplane. Notably, all four images showed a very distinct bright metallic sheen, which again supported the successful adsorption of AgNP on BNNS.

**Figure 3:**
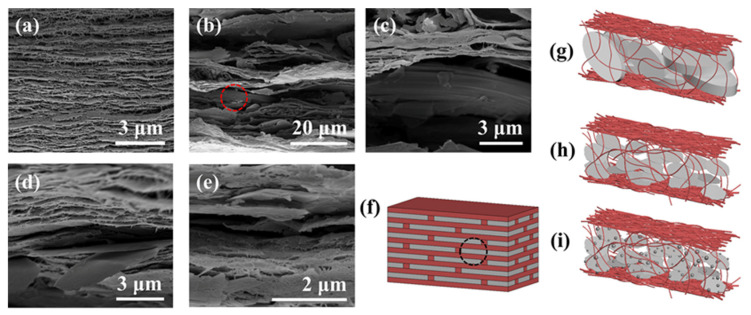
SEM images of the fractured surfaces of (**a**) pure ANF, (**b**) ANF/h-BN, (**c**) SEM image with higher magnification corresponding to (**b**). (**d**) ANF/BNNS, and (**e**) ANF/BNNS@Ag thermally conductive composite films. (**f**) Schematic diagrams of the structure of thermally conductive composite films. Schematic diagrams of the structure of magnification of (**g**) ANF/h-BN, (**h**) ANF/BNNS, and (**i**) ANF/BNNS@Ag conductive composite films.

Due to the interface interaction between ANF and fillers, and the accumulation of top-down vacuum filtration, the thermal conductive composite films presented a stacked layered “brick and mortar” structure (Figure 3f). Therein, bricks were made of various thermal conductive fillers, and the ANF as mortar was twisted and interwoven with the former. Figure 3g–i shows the structural characteristics of three kinds of thermal conductive composite films. The “bricks” in the ANF/h-BN film were relatively large and thick, which led to an intermittent heat conduction path (Figure 3g) and could not form an effective heat conduction network. The “bricks” in the ANF/BNNS and ANF/BNNS@Ag films had ultra-thin thicknesses and large specific surface areas, which was conducive to the formation of continuous thermal conduction paths (Figure 3h,i). These inferences were further confirmed by SEM images of the fractured surfaces of composite films (Figure 3a–e). Moreover, h-BN, BNNS, and BNNS@Ag were all arranged in the horizontal direction with a high degree of in-plane orientation. Air voids were used as an index to evaluate the binding effect of filler and polymer matrix [70,71]. The fractured surface of ANF/h-BN film was coarser than those of other composite films, which was attributed to the larger volume of unexfoliated h-BN, and thus the interface interaction between ANF and h-BN was weaker than that of the other two composite films, resulting in the weak connection between h-BN and ANF (Figure 3b,c). Then ANF/BNNS (Figure 3d) and ANF/BNNS@Ag (Figure 3e) composite films had no large voids and other obvious interfacial debonding, which was favorable for reducing the interfacial thermal resistance between fillers and ANF, and improved phonon transport efficiency. In addition, the fillers in ANF/BNNS and ANF/BNNS@Ag films had good uniformity and dispersion, and no agglomeration of fillers was found, which could be attributed to the good affinity between fillers and matrix, and strong interfacial bonding strength in two kinds of films. Moreover, in ANF/BNNS@Ag films, AgNP existed on the surface of BNNS, which made it easy to form bridging channels between adjacent BNNS@Ag films, thus improving the heat conduction rate. As shown in Supplementary Materials, Figure S11, low-magnification SEM images of the fractured surfaces of composite films could estimate the thickness of the four composite films to be about 30 μm, 110 μm, 90 μm, and 80 μm, respectively. It could be attributed to the addition of fillers, which increased the thickness of the film without changing the base area. Note that the thickness of ANF/BNNS and ANF/BNNS@Ag films was smaller than that of ANF/h-BN films. This was because BNNS and BNNS@Ag were smaller in volume, thinner in thickness, and larger in surface area than h-BN films. Therefore, they were more closely connected with ANF and had stronger interface interaction. It further confirmed that no agglomeration occurs in the fillers of ANF/BNNS film and ANF/BNNS@Ag film. The existence of AgNP was clearly confirmed by the results of the energy dispersive spectrometer (EDS) spectrum, as shown in Supplementary Materials, Figure S12a,b. The peaks of B (0.18 keV), C (0.28 keV), N (0.39 keV), O (0.53 keV), and Ag (2.64 keV and 2.99 keV) are shown in the EDS spectrum, and the two peaks of Ag correspond to the two Ag peaks shown in the XPS spectra of BNNS@Ag in Figure S5 (Supplementary Materials), respectively, which proved the existence of AgNP in the film. The SEM image corresponding to Figure S12a (Supplementary Materials) is shown in Figure S12c (Supplementary Materials), and Figure S12d–h (Supplementary Materials) shows that all elements are uniformly distributed without aggregation. Especially, BNNS@Ag was uniformly distributed in the film, which was beneficial to improve the thermal conductivity of the composites.

### 3.3. Thermal Conductivity of Composite Films

**Figure 4:**
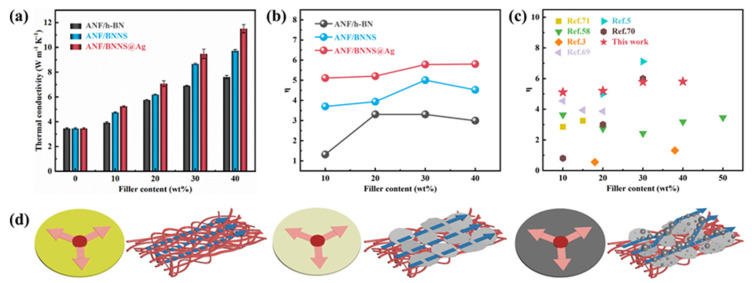
(**a**) Thermal conductivity of the different composite films. (**b**) Thermal conductivity enhancement factors of the different composite films. (**c**) Comparison of thermal conductivity enhancement factors of this work and other reports. Schematic diagrams of thermal conducting models in the (**d**) pure ANF, ANF/BNNS, and ANF/BNNS@Ag composite films.

Variation of in-plane thermal conductivities of different composite films with increasing filler content are shown in Figure 4a. It can be seen from Figure 4a that with the increase of filler content, the thermal conductivity of the three composite films was gradually increasing, which could be attributed to the good interaction between fillers and ANF, which reduced phonon scattering and was conducive to phonon propagation. In addition, the main chain of the ANF molecular chain contained a symmetrical benzene ring structure, which was conducive to the spontaneous formation of directional and orderly layered structures with fillers during vacuum filtration, so that phonons could be efficiently transmitted along the layers where the main chain was located, forming a continuous and stable thermal conduction path. The specific heat capacity, the in-plane thermal diffusivity, the density, the thermal conductivity, the increased percentage, and the enhancement factor are shown in Supplementary Materials, Table S1. The thermal conductivity of ANF/h-BN, ANF/BNNS, and ANF/BNNS@Ag films with filler content of 40 wt.% were 7.61 W m^−1^ K^−1^, 9.73 W m^−1^ K^−1^, and 11.51 W m^−1^ K^−1^, respectively, which were increased by 120.49%, 181.85%, and 233.27% compared with pure the ANF film, respectively. Hence, the addition of fillers significantly improved the thermal conductivity of the composites, and the best improvement effect was BNNS@Ag.

In order to further explain the influence of fillers on the thermal conductivity of composites and accurately describe the ability of fillers to improve the thermal conductivity, the thermal conductivity enhancement efficiency η was defined and calculated according to the following Equation (3):

(3) $$\eta =\frac{\lambda_{c}-\lambda_{m}}{\varphi_{f}\lambda_{m}}$$

where λ_c_ is the thermal conductivity of the composite material, λ_m_ is the thermal conductivity of the matrix, and φ_f_ is the content of the filler. In the ANF/h-BN film, the thermal conductivity of h-BN was still improved to a certain extent due to its very high thermal conductivity and sufficient filler content, which still formed a certain number of thermal conductivity paths. Then in the ANF/BNNS film, due to the ultra-thin BNNS, large specific surface area, and long radial length, the probability of BNNS contact stacking was higher and the thermal conduction path was easier to form. Moreover, there were fewer voids in the films, which reduced the thermal resistance at the interface and the phonon scattering, thus leading to a higher increase in the thermal conductivity of ANF/BNNS films. Furthermore, in the ANF/BNNS@Ag film, AgNP repaired the defect of BNNS and filled the voids between the neighboring BNNS in contact with each other, which further reduced the interface thermal resistance. The BNNS@AgNP–BNNS connection had a good synergistic effect, which helped to build interconnected thermal conduction paths, thus effectively promoting the rapid transmission of phonons. Therefore, with the same filler content, the thermal conductivity of the ANF/BNNS@Ag film was higher. As seen in Figure 4b, the enhancement factors of ANF/BNNS and ANF/BNNS@Ag films decreased when the fillers content increased from 30 wt.% to 40 wt.%, while that of ANF/h-BN films even began to decrease when the fillers content increased from 20 wt.% to 30 wt.%. This could be attributed to the overabundance of fillers, which resulted in the increase of cavities and voids, additional phonon scattering, and a decrease in efficiency factor. Note that the enhancement factor of ANF/h-BN film decreased in advance, which proved that the affinity between h-BN and ANF was weaker than that of BNNS and BNNS@Ag. Figure 4c shows the thermal conductivity enhancement factors (η) in this work compared with other reports [3,6,61,72,73,74]. This could be attributed to the formation of the “brick and mortar” structure and the “BNNS–AgNP–BNNS” bridge in the ANF/BNNS@Ag film, which could form higher density thermal conductivity paths. Models of thermal conducting paths of pure ANF, ANF/BNNS, ANF/BNNS@Ag films are proposed in Figure 4d–f. The pure ANF film conducted heat through phonons generated by the vibration of atoms, groups, and molecular chains, that is, it completely depends on the thermal conductivity of ANF itself. Through the uniform dispersion of BNNS, ANF/BNNS film formed a thermal conductivity path in the film, and the thermal conductivity of the composite was improved. Furthermore, the thermal conductivity of BNNS@Ag composites was improved better, which could be attributed to the following three points: First, the intrinsic thermal conductivity of AgNP was greater than that of BNNS. Secondly, the AgNP filling between BNNS and adjacent BNNS was equivalent to the repair and extension of the thermal conductive path, thus forming a more effective thermal conductive network. In addition, it could be related to the interface interaction between BNNS@Ag and ANF being stronger than that between BNNS and ANF, which reduced the interfacial thermal resistance.

### 3.4. Other Properties of Composite Films

**Figure 5:**
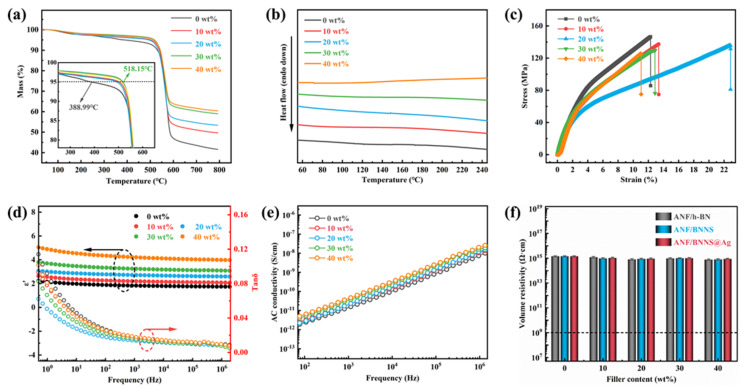
(**a**) TGA curves, (**b**) DSC curves, (**c**) stress-strain curves, (**d**) frequency dependence curves of dielectric constant and dielectric loss tangent, and (**e**) AC conductivity curves of pure ANF and ANF/BNNS@Ag films with various contents. (**f**) Volume resistivity of the different composite films with various filler contents.

The TGA curves were applied to analyze thermostability of composite films. As shown in Figure 5a, the mass of all kinds of films was relatively stable before 500 °C, and the TGA curves basically coincided. The initial decomposition temperature of ANF/BNNS@Ag films was significantly higher than that of pure ANF film, and the highest temperature reached 518.15 °C. After 500 °C, the TGA curves of all the films dropped sharply, but still coincided, showing a similar one-step decomposition form. After 600 °C, the higher the filler content, the higher the residual weight, which could be attributed to the high thermal stability of BNNS, which improved the thermal stability of composite films, which was of great significance for thermal conductivity composites working at high temperatures. In addition, it can be seen from Figure 5b that the glass transition temperature of pure ANF film itself was not obvious, while the addition of BNNS@Ag had little influence on the melting behavior of ANF, which further proved that the addition of BNNS@Ag had not weakened the thermal stability of ANF. Figure 5c shows the stress-strain curves of pure ANF and ANF/BNNS@Ag films with various filler contents, and the corresponding storage modulus is shown in Supplementary Materials, Table S2. The tensile strength of pure ANF film reached 146.62 MPa. With the increase of BNNS@Ag content, the tensile strength gradually decreased, and when the content of BNNS@Ag was 40 wt.%, the tensile strength decreased to 129.14 MPa. Moreover, both pure ANF and ANF/BNNS@Ag films exhibited nonlinear deformation behavior with brittleness characteristics and different elongations at breaking point. This could be attributed to the fact that ANF could be closely connected by forces such as hydrogen bonds, which was the reason for its high tensile strength. However, the higher the content of BNNS@Ag, the more it was likely to produce the contact interface with ANF in the film and then produce mechanical defects and stress concentration points. However, the mechanical properties of ANF/BNNS@Ag films were still relatively good, and they were still suitable for most heat dissipation occasions.

The frequency dependence of the dielectric constant and dielectric loss tangent of pure ANF and ANF/BNNS@Ag films are shown in Figure 5d. The results showed that the dielectric constant of ANF/BNNS@Ag composite films had a weak frequency dependence. With the increase of BNNS@Ag content, the dielectric constant of the composite gradually increased. When the frequency was 10^6^ Hz, the dielectric constant of the composite with 40 wt.% content was about 3.99, which was about 1.3 times higher than that of pure ANF (~1.75). This could be attributed to the fact that after the addition of fillers in the polymer, due to the effect of interface polarization, the charge accumulation at the interface formed a micro capacitor, which increased the storage of electric energy and improved the dielectric constant of the composite material [30]. Moreover, AgNP increased the conductivity difference between BNNS and ANF. The larger conductivity difference increased the space charge polarization and polarization reverse velocity, which also increased the interface polarization effect, that is, the Maxwell–Wagner–Sillars (MWS) effect [75]. However, the dielectric loss was strongly dependent on frequency. The results showed that all the composite films had very low dielectric loss, which was mainly due to the low tanδ of ANF and BNNS. In the low frequency region, the dielectric loss dropped sharply, which could be attributed to the interface polarization. At 50 Hz, the highest dielectric loss of the film was 0.03, which was pure film. In the high frequency operating range of 10^4^–10^6^ Hz, the dielectric loss was almost no longer reduced; it’s a total reduction of 0.008. Moreover, it is worth noting that the dielectric loss decreased with the increase of BNNS@Ag content and reached the minimum value at 20 wt.%. This was because the tanδ of BNNS@Ag was smaller than that of ANF, and the filler filled the voids between ANF and reduced defects at a lower addition content, thus reducing the dielectric loss. Then dielectric loss gradually increased. This could be attributed to the fact that the addition of the filler would introduce carriers, which would lead to the increase of leakage current and then lead to the increase of dielectric loss. In addition, with the further increase of filler, the void in the film would not reduce, but gradually increase, leading to the increase of dielectric loss. When working at high frequencies, the polarization of dipole and space charge was very weak or non-existent, and the defects in the composites became an important factor affecting the dielectric loss [76]. However, when the filler content was 40 wt.%, its tanδ was still less than that of pure ANF film, which was due to the Coulomb block and quantum confinement effect of AgNP [77,78]. Figure 5e shows that with the increase of BNNS@Ag content, the AC conductivity of the composite increased slightly. At 10^6^ Hz, the AC conductivity of pure ANF film was 8.737 × 10^−9^ S/cm, while that of ANF/BNNS@Ag 10 wt.% film was 1.061 × 10^−8^ S/cm. The slight increase of the AC conductivity indicated that even if there was AgNP in the composite, no effective conductive path was formed, and the insulation property of the material almost did not decrease. All of them were about 10^15^ Ω·cm and still had excellent electrical insulation to meet the requirements of insulating equipment (Figure 5f). It was consistent with the insulation performance shown in Figure 2e. This could be attributed to the inherent nature of BNNS and ANF themselves with volume resistivity of 1.1 × 10^16^ Ω·cm and 4.3 × 10^14^ Ω·cm, respectively [79].

### 3.5. Simple Applications of Composites

**Figure 6:**
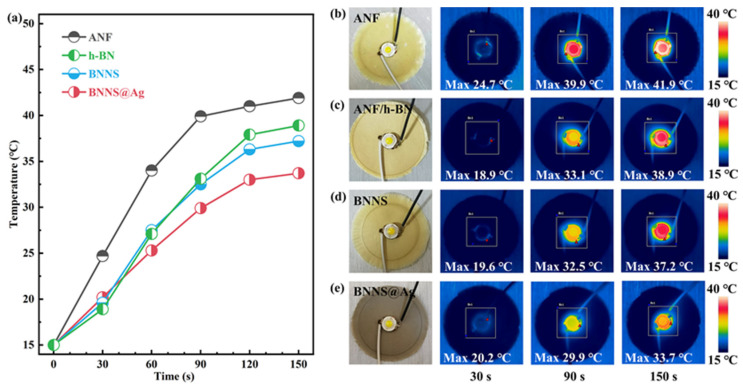
(**a**) The curves of surface temperature variation of the different composite films as a function of time upon heating. (**b**–**e**) Infrared thermal images of the different composite films.

**Figure 7:**
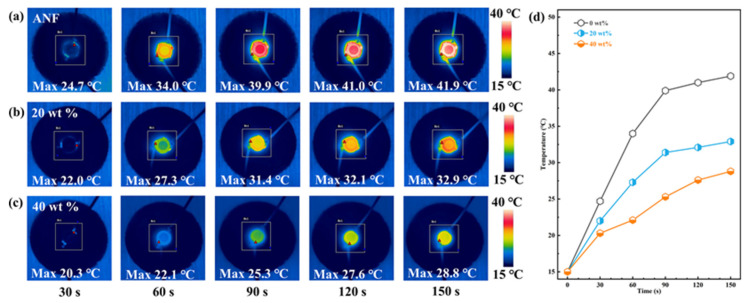
(**a**–**c**) Infrared thermal images of thermal distribution of the ANF/BNNS@Ag films with various filler contents under LED heat. (**d**) The curves of surface temperature variation of the ANF/BNNS@Ag films with various filler contents as a function of time upon heating.

Moreover, in order to evaluate the thermal management capability of the thermal conductive composite, we recorded the surface temperature changes of the composite films under the operation of a light emitting diode (LED) with an infrared thermal imager, in which the LED was adhered to the center of the film with a thermal conductive copper adhesive. When the LED worked, it would become the heating center, and the heat dissipation rate of the film could reflect the thermal management capability of the film. The temperature distribution image of the film and the corresponding surface temperature–time change curve are shown in Figure 6 and Figure 7, respectively. Figure 6 shows the surface temperature changes of pure ANF, ANF/h-BN, ANF/BNNS, and ANF/BNNS@Ag film, in which the mass fraction of composite film filler was 10%. It could be seen from Figure 6a that the temperature of pure ANF film rose the fastest, and the maximum temperature at each time point was much higher than that of the three composites, which indicated that the addition of fillers indeed improved the thermal conductivity of the composites. The temperature tended to be stable after 120 s, and the film with the lowest temperature was ANF/BNNS@Ag film, indicating that ANF/BNNS@Ag film had the best heat dissipation performance among the three composites, which further proved that the thermal conductivity was the highest.

Figure 7d shows that with the increase of BNNS@Ag content, the heat dissipation performance of the composite film was gradually improved. The temperature–time curve remained stable after 120 s. The lowest temperature of ANF/BNNS@Ag 40 wt.% film after stabilization was 28.8 °C, while the highest temperature of ANF film was 41.9 °C. In general, the temperature–time variation of various films was consistent with the thermal conductivity variation shown in Figure 3a.

### 3.6. Finite Element Analysis

**Figure 8:**
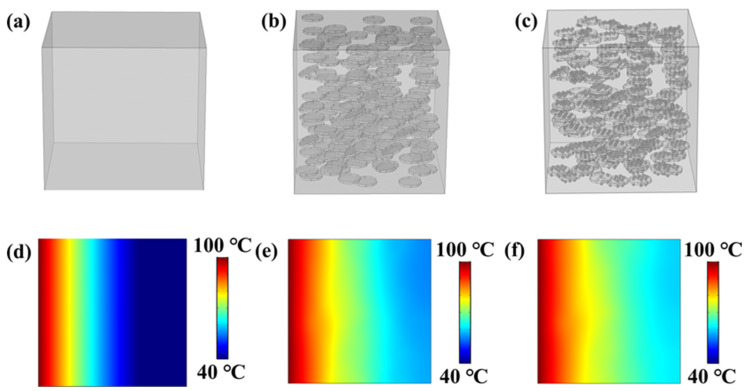
Finite element simulation of the variation of heat transfer of (**a**,**d**) the pure ANF, (**b**,**e**) ANF/BNNS, and (**c**,**f**) ANF/BNNS@Ag nanocomposites.

In order to clarify the effect of BNNS@Ag filler on the thermal conductivity of ANF matrix, the transient temperature distributions of pure ANF, ANF/BNNS nanocomposites, and ANF/BNNS@Ag nanocomposites were simulated by using the finite element simulation method. As shown in Figure 8a–c, the pure ANF model was established as a cube of 1 mm × 1 mm × 1 mm. On this basis, BNNS were added to establish the ANF/BNNS model. AgNP was further added to establish the ANF/BNNS@Ag model. It was assumed that heat flow was uniformly transmitted in the composite material, and the temperature on the left side was set to 100 °C. In addition, all surfaces except the right and left sides were considered to be adiabatic. The material parameters were as follows: thermal conductivity, density, and specific heat capacity of BNNS were respectively set as 300 W m^−1^ K^−1^ [12,13], 2.25 g/cm^3^, and 0.73 kJ/(kg·°C) [80]. The thermal conductivity, density, and specific heat capacity of Ag were set as 490 W m^−1^ K^−1^ [81], 10.49 g/cm^3^, and 0.24 kJ/ (kg·°C), respectively. According to Table S1 (Supplementary Materials), the thermal conductivity, density, and specific heat capacity of ANF were set as 3.4528 W m^−1^ K^−1^, 1.1109 g/cm^3^, and 1.5395 kJ/ (kg·°C), respectively. In addition, the mass fraction of BNNS in the ANF/BNNS@Ag nanocomposite model was 19.46 wt.%, and the mass fraction of BNNS@Ag in the ANF/BNNS@Ag nanocomposite model was 19.44 wt.%, and the filler was roughly uniform. The temperature distribution of the three models is shown in Figure 8d–f. It can be seen that the lowest temperature on the right side was pure ANF, followed by ANF/BNNS nanocomposites, and the highest temperature was ANF/BNNS@Ag nanocomposites. The simulation result showed that the thermal conductivity of ANF/BNNS@Ag nanocomposites was better than that of pure ANF and ANF/BNNS nanocomposites, which further proved that the “BNNS–AgNP–BNNS” thermal conductivity path was better than the BNNS thermal conductivity path. Therefore, the BNNS@Ag filler formed by attaching AgNP to the surface of BNNS could greatly improve the thermal conductivity of the ANF matrix.

## 4. Conclusions

The work was undertaken to design a simple but effective strategy for preparing BNNS@Ag. Under ultrasonic and hydrothermal conditions, the liquid-phase exfoliation of h-BN with Na^+^ was performed. The yield of BNNS obtained was close to 50%, the number of layers was small, and no obvious agglomeration occurred. At the same time, the in situ reduction of citrate was used to reduce Ag^+^ to produce Ag, which was deposited on the surface of BNNS to obtain BNNS@Ag. In this work, trisodium citrate had both exfoliation and reduction effects. The obtained BNNS@Ag had a complete crystal structure, and the uniform distribution of AgNP on the surface could be clearly observed by SEM and TEM, and the agglomeration still did not occur. Then a series of composites with different filler contents was prepared by using BNNS@Ag as filler. The results showed that the thermal conductivity of the ANF/BNNS@Ag composite with a filler content of 40 wt.% was 11.51 W m^−1^ K^−1^, which was 233.27% higher than that of pure ANF. Therefore, we believe that AgNP acts as a thermal “bridge” between BNNS, thus making the “brick-and-mortar” structure more compact and orderly. This provides phonon conduction paths and reduces interfacial thermal resistance. In addition, ANF/BNNS@Ag composites still maintained high tensile strength, high volume resistivity, low dielectric constant, and low dielectric loss in a reasonable range. Hence, BNNS@Ag is considered as a new thermal conductive filler that can improve the thermal conductivity of polymer while maintaining electrical insulation. In summary, it is feasible to prepare BNNS@Ag by using trisodium citrate to achieve efficient exfoliation of h-BN and in situ reduction of Ag, and the interface thermal resistance of BNNS@Ag is effectively reduced compared with that of BNNS. Moreover, this article has provided deeper insights into exfoliation and functionalization of other two-dimensional sheet materials, which gained from this study may be of assistance for the efficient preparation of other high thermal conductivity fillers.

## Data Availability

The data presented in this study are available upon request from the corresponding author.

## Supplementary Materials

The following are available online at https://www.mdpi.com/article/10.3390/polym13132028/s1, Figure S1: (a) Comparison of the stability of h-BN and BNNS. (b) The exfoliated BNNS in different ratios of H2O and IPA with different types of alkali metal ions. Figure S2: (a) SEM images of h-BN and (b) the corresponding side enlargement. (c) SEM image of BNNS. (d) TEM image of BNNS. Figure S3: (a) The complete XRD patterns of h-BN and BNNS exfoliated by Li^+^, Na^+^, K^+^, and pure solvent. (b) Raman spectra of h-BN and BNNS. Figure S4: TEM images of (a) BNNS@Ag, and (b) the partial magnification of BNNS@Ag. XRD patterns of (c) BNNS@Ag, and (d) the magnification of characteristic peaks. Figure S5: XPS spectra of (a) BNNS@Ag, and (b) the corresponding peak magnification of Ag. Figure S6: (a) FT-IR spectra of the BNNS and BNNS@Ag. (b) TGA curves of the h-BN, BNNS, and BNNS@Ag. Figure S7: Tyndall effect of the BNNS and BNNS@Ag. Figure S8: SEM image of Kevlar yarn. Figure S9: Photographs of (a) ANF aqueous solution, (b) BNNS@Ag aqueous solution, and (c) ANF/BNNS@Ag mixed solution of after high shear. (d) Schematic diagram of mixed solution of ANF and BNNS@Ag. Figure S10: Photograph of pure ANF film, 30 wt.% ANF/h-BN, 30 wt.% ANF/BNNS, and 30 wt.% ANF/BNNS@Ag thermally conductive composite films. Figure S11: Low-magnification SEM images of the fractured surfaces of (a) pure ANF, (b) ANF/h-BN, (c) ANF/BNNS, and (d) ANF/BNNS@Ag thermally conductive composite films. Figure S12: (a) EDS elements mapping, and (b) the corresponding EDS spectrum of the ANF/BNNS@Ag film. (c) SEM image of ANF/BNNS@Ag film corresponding to (a), and corresponding EDS elements mapping images of (d) C, (e) N, (f) Ag, (g) B, and (h) O. Table S1: The density, the specific heat capacity, the thermal diffusivities, thermal conductivity, the growth rate relative to the pure ANF film, and thermal conductivity enhancement factors of the pure ANF, ANF/BNNS, and ANF/BNNS@Ag composite films. Table S2: Storage modulus of pure ANF and ANF/BNNS@Ag films with various contents corresponding to Figure 5c.
