# Supplementary material for: Ultra-Robust Thermoconductive Films Made from Aramid Nanofiber and Boron Nitride Nanosheet for Thermal Management Application
Source: Polymers (Basel). 2021 Jun 22;13(13):2028. doi: 10.3390/polym13132028 (PMC8271841; doi:10.3390/polym13132028)
Supplement: Supplementary file 1 [file polymers-13-02028-s001.zip › polymers-1238605-supplementary.pdf]

Supplementary Materials

Robust Biomimetic Nacreous Aramid Nanofiber/Boron Nitride Nanosheet Films with Excellent Thermal Management Properties

Li-Hua Zhao [1], Yun Liao [1], Li-Chuan Jia [1], Zong-Xi Zhang [2], Zhong Wang [1], Xiao-Long Huang [1], Wen-Jun Ning [1], Jun-Wen Ren [1,*]

[1] *College of Electrical Engineering, Sichuan University, Chengdu 610065, China*

[2] *State Grid of China, State Grid Sichuan Electric Power Research Institute, Chengdu 610041, China*

*Correponding author.

E-mail address: myboyryl@scu.edu.cn (Junwen Ren)

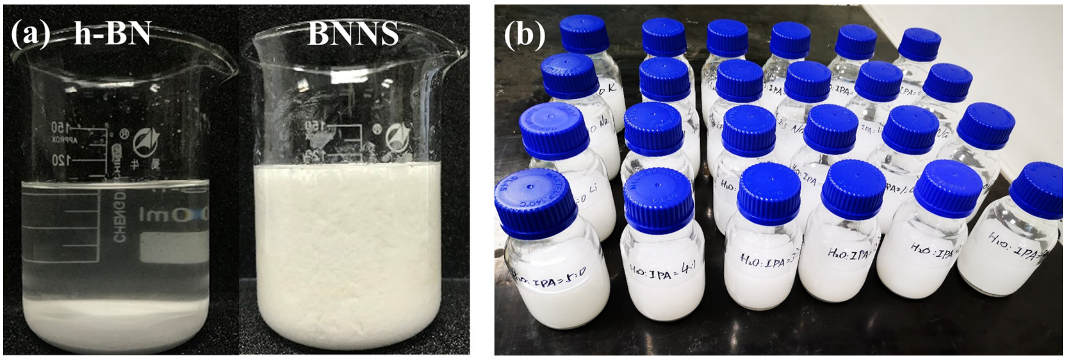

Figure S1. (**a**) Comparison of the stability of h-BN and BNNS. (**b**) The exfoliated BNNS in different ratios of H_2O and IPA with different types of alkali metal ions.

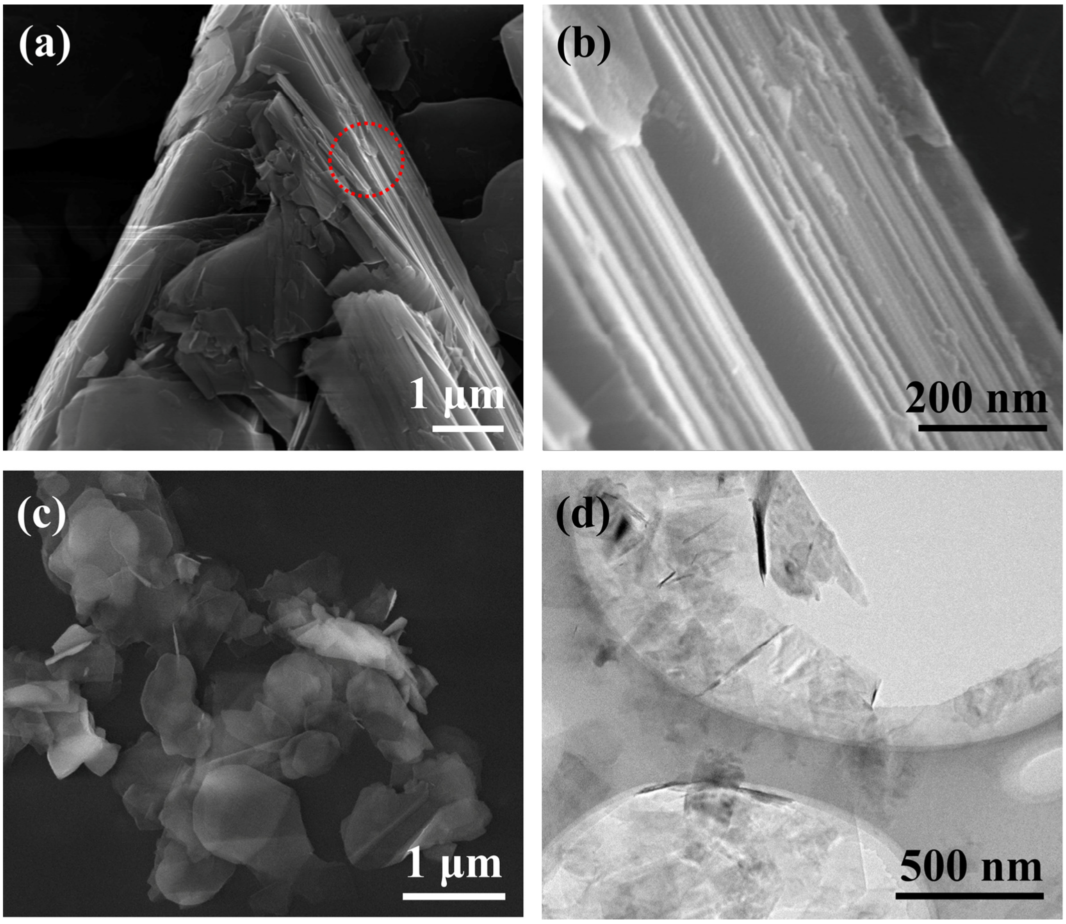

Figure S2. (**a**) SEM images of h-BN and (**b**) the corresponding side enlargement. (**c**) SEM image of BNNS. (**d**) TEM image of BNNS.

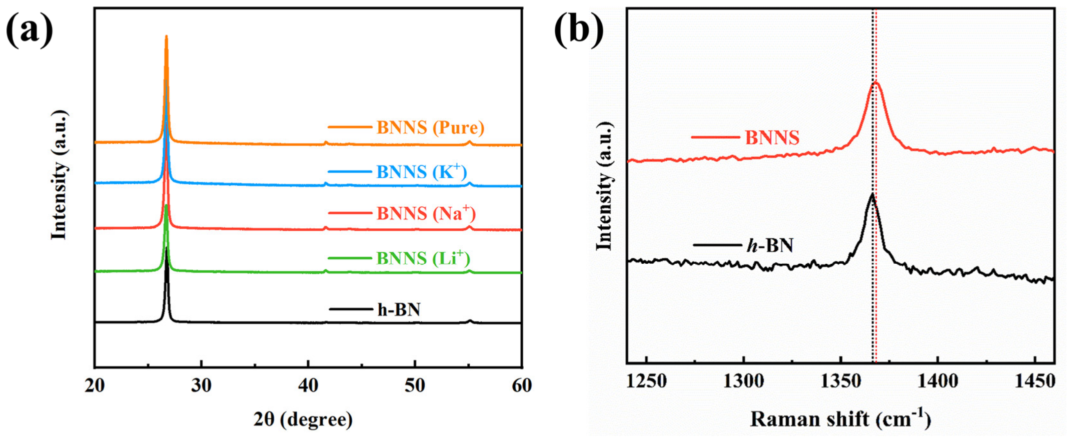

Figure S3. (**a**) The complete XRD patterns of h-BN and BNNS exfoliated by Li^+, Na^+, K^+, and no ions. (**b**) Raman spectra of h-BN and BNNS.

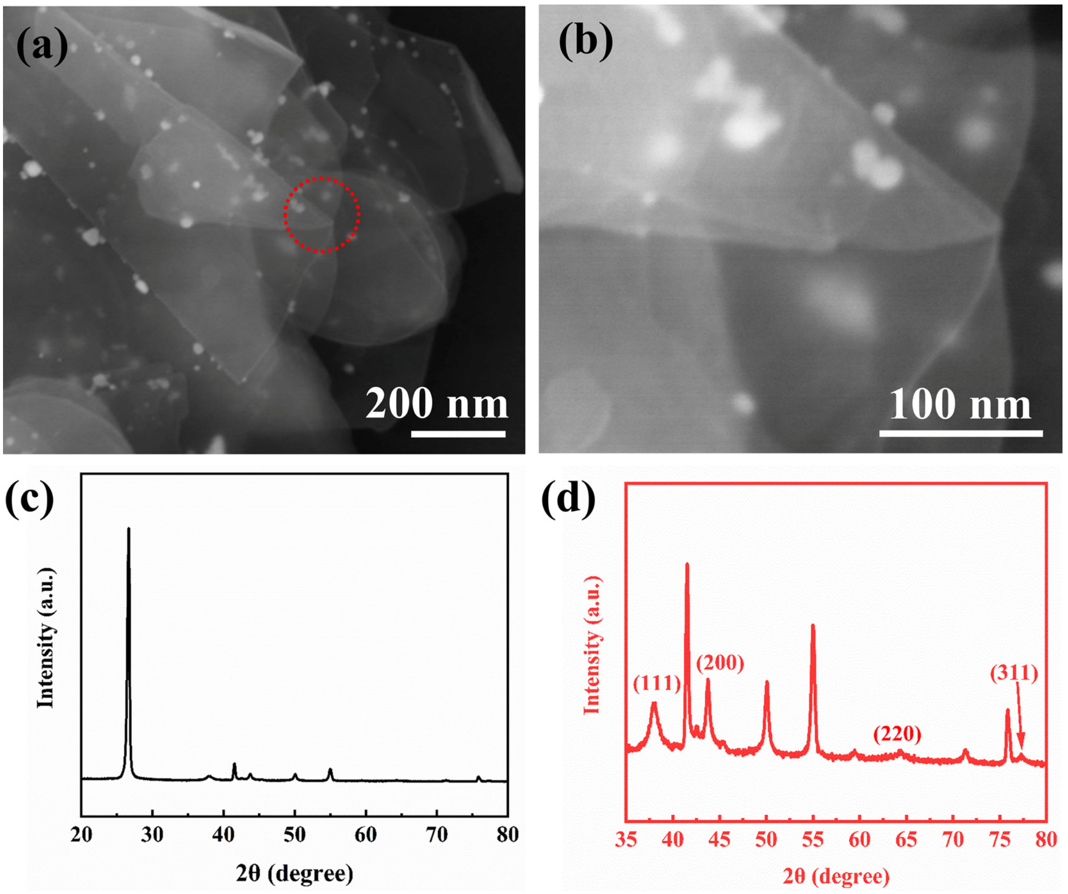

Figure S4. TEM images of (**a**) BNNS@Ag, and (**b**) the partial magnification of BNNS@Ag. XRD patterns of (**c**) BNNS@Ag, and (**d**) the magnification of characteristic peaks.

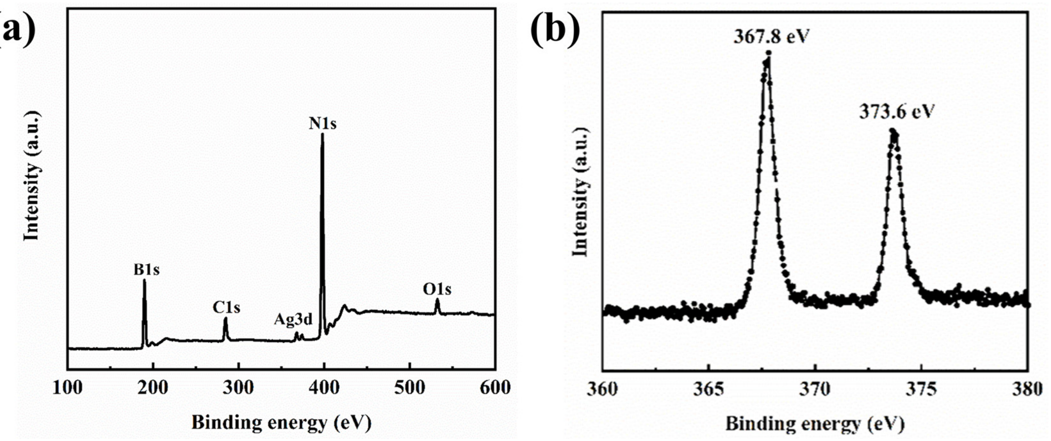

Figure S5. XPS spectra of (**a**) BNNS@Ag, and (**b**) the corresponding peak magnification of Ag.

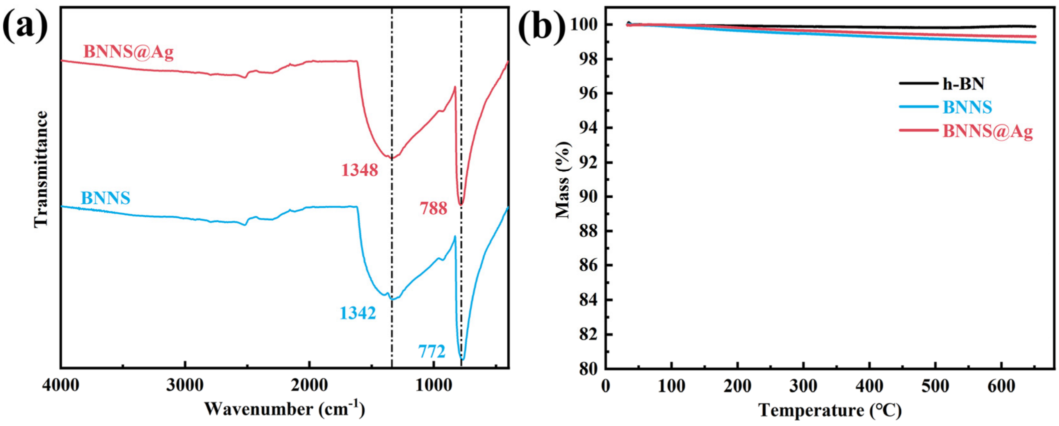

Figure S6. (**a**) FT-IR spectra of the BNNS, and BNNS@Ag. (**b**) TGA curves of the h-BN, BNNS, and BNNS@Ag.

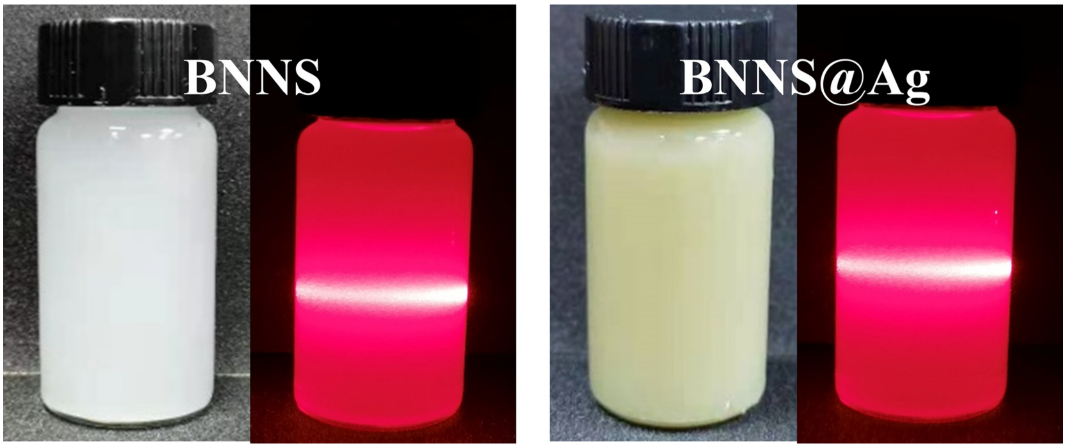

Figure S7. Tyndall effect of the BNNS and BNNS@Ag.

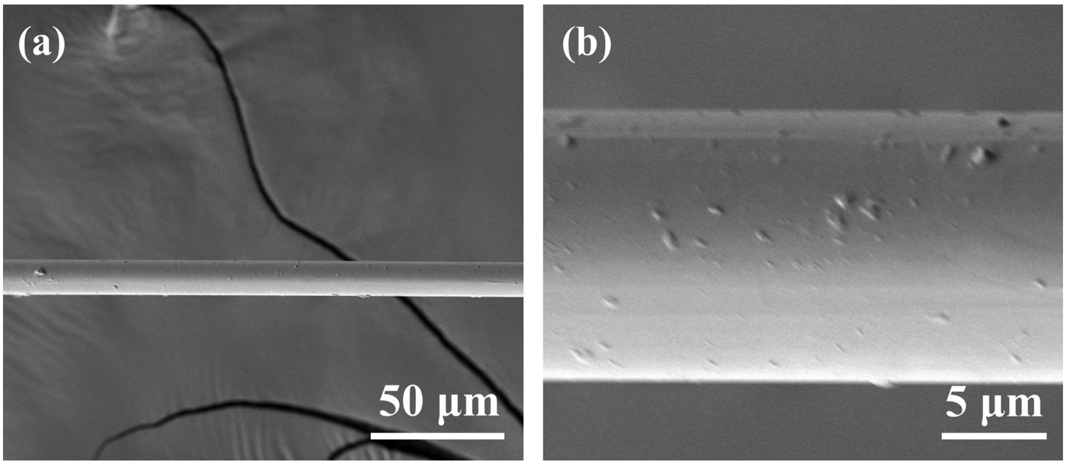

Figure S8. SEM image of Kevlar yarn.

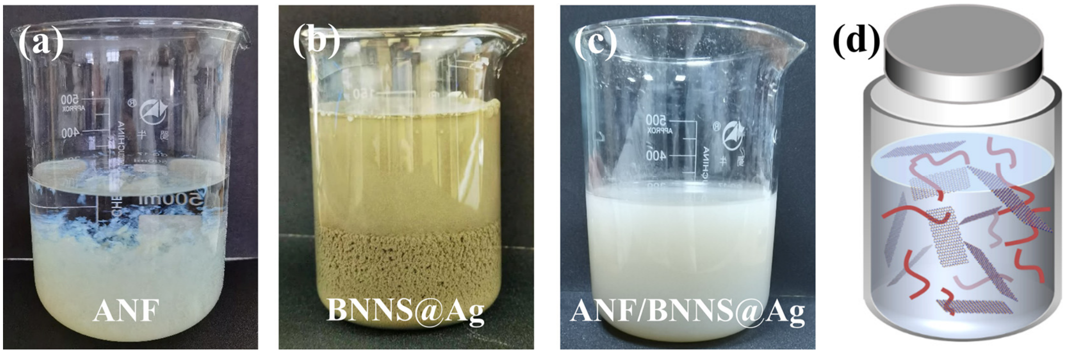

Figure S9. Photographs of (**a**) ANF aqueous solution, (**b**) BNNS@Ag aqueous solution, and (**c**) ANF/BNNS@Ag mixed solution of after high shear. (**d**) Schematic diagram of mixed solution of ANF and BNNS@Ag.

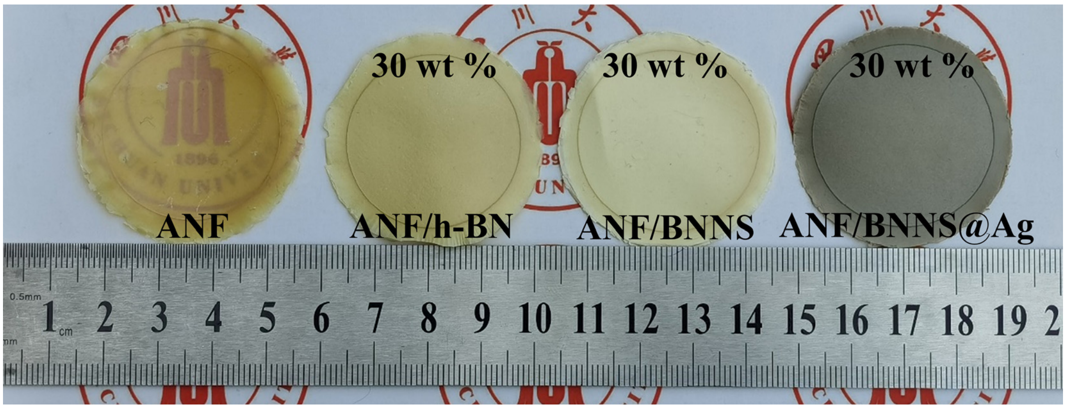

Figure S10. Photograph of pure ANF film, 30 wt.% ANF/h-BN, 30 wt.% ANF/BNNS, and 30 wt.% ANF/BNNS@Ag thermally conductive composite films.

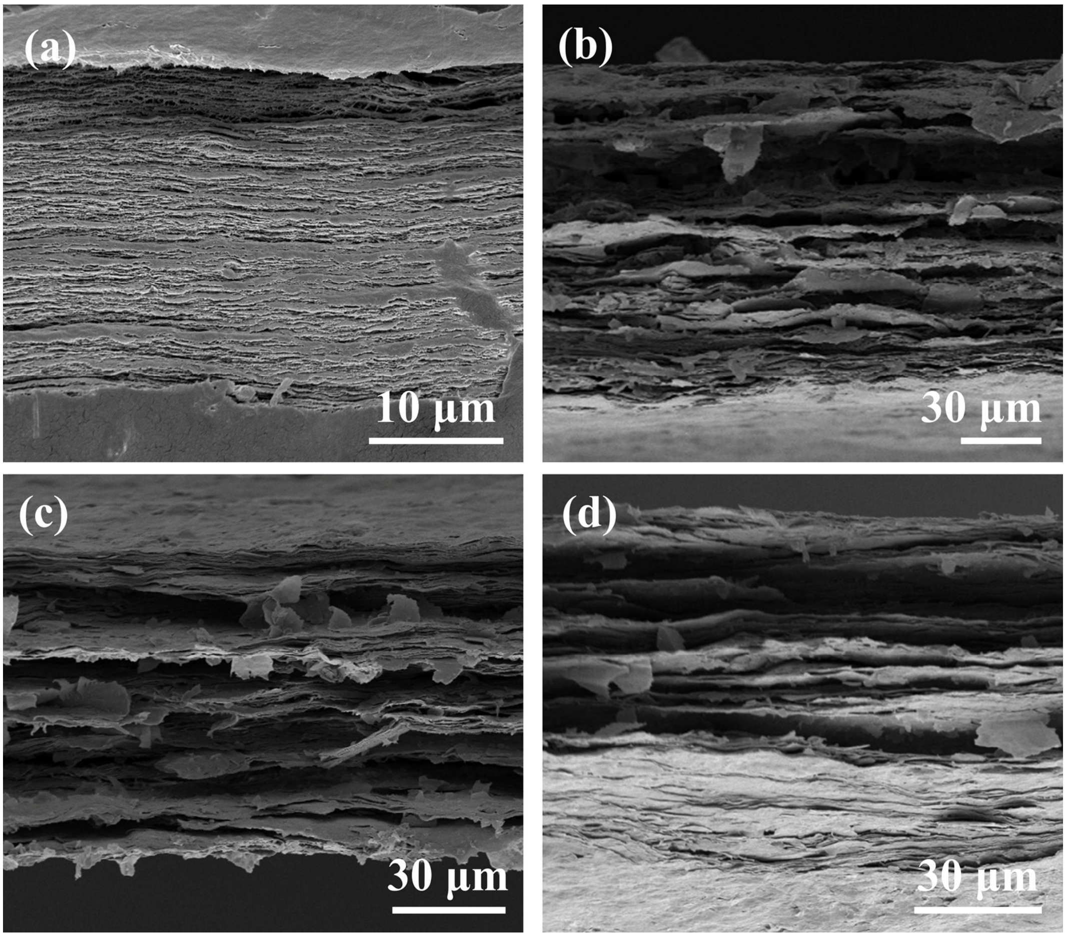

Figure S11. Low-magnification SEM images of the fractured surfaces of (**a**) pure ANF, (**b**) ANF/h-BN, (**c**) ANF/BNNS, and (**d**) ANF/BNNS@Ag thermally conductive composite films.

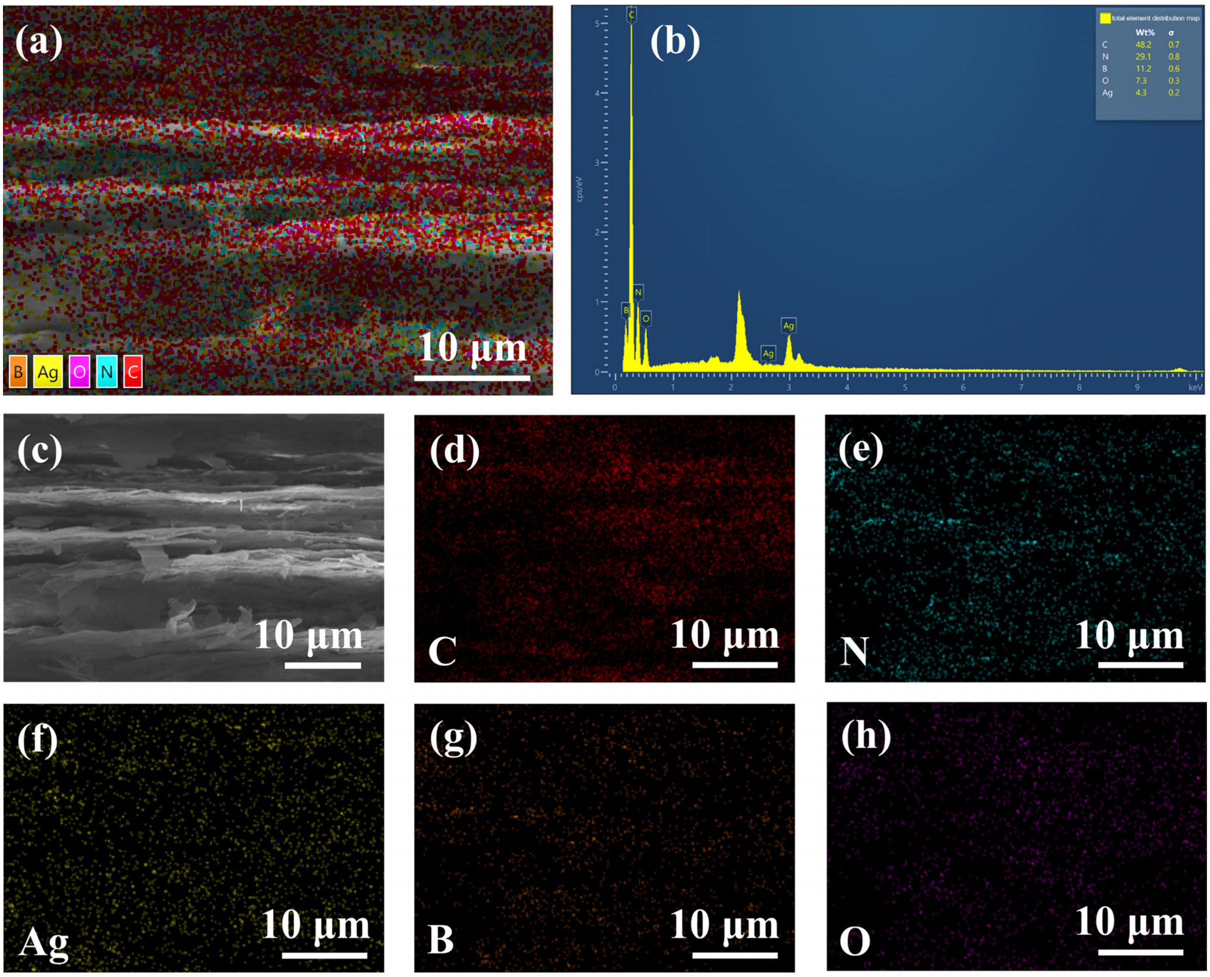

Figure S12. (**a**) EDS elements mapping, and (**b**) corresponding the EDS spectrum of the ANF/BNNS@Ag film. (**c**) SEM image of ANF/BNNS@Ag film corresponding to (**a**), and corresponding EDS elements mapping images of (**d**) C, (**e**) N, (**f**) Ag, (**g**) B and (**h**) O.

Table S1. The density, the specific heat capacity, the thermal diffusivities, thermal conductivity, the growth rate relative to the pure ANF film, and thermal conductivity enhancement factors of the pure ANF, ANF/BNNS, and ANF/BNNS@Ag composite films.

	**Specific heat capacity (J/g·℃)**	**In-plane thermal diffusivity (mm^2/s)**	**Density (g/cm^3)**	**Thermal conductivity ($W\ m^{-1}\ K^{-1}$)**	**Increased percentage (%)**	**Enhancement factor (η)**
**Pure ANF**	1.53	2.03	1.11	3.45	0	1
**ANF/h-BN 10 wt.%**	1.36	2.45	1.18	3.92	13.53	1.31
**ANF/h-BN 20 wt.%**	1.44	3.16	1.26	5.76	66.69	3.31
**ANF/h-BN 30 wt.%**	1.38	3.91	1.28	6.90	99.81	3.30
**ANF/h-BN 40 wt.%**	1.25	4.42	1.37	7.61	120.49	2.99
**ANF/BNNS 10 wt.%**	1.37	3.02	1.15	4.75	37.45	3.70
**ANF/BNNS 20 wt.%**	1.26	3.84	1.28	6.19	79.35	3.94
**ANF/BNNS 30 wt.%**	1.40	5.05	1.23	8.67	150.97	5.00
**ANF/BNNS 40 wt.%**	1.22	6.88	1.16	9.73	181.85	4.52
**ANF/BNNS@Ag 10 wt.%**	1.20	3.43	1.28	5.23	51.61	5.11
**ANF/BNNS@Ag 20 wt.%**	1.28	3.93	1.40	7.07	104.77	5.20
**ANF/BNNS@Ag 30 wt.%**	1.32	5.68	1.27	9.47	174.38	5.78
**ANF/BNNS@Ag 40 wt.%**	1.19	7.54	1.29	11.51	233.27	5.80

Table S2. Storage modulus of pure ANF and ANF/BNNS@Ag films with various contents corresponding to **Figure 5c**.

**Content (%)**	0	10	20	30	40
**Storage modulus (Mpa)**	1.53	2.03	1.11	3.45	0
